# Prospects for tuberculosis elimination in Ethiopia: feasibility, challenges, and opportunities

**DOI:** 10.11604/pamj.2022.43.146.35557

**Published:** 2022-11-17

**Authors:** Tefera Belachew Agizew, Zewdu Gashu Dememew, Taye Leta, Nebiyu Hiruy, Emawayish Tesema, Eshetu Abdissa Abelti, Asfawesen Gebreyohannes, Yohannes Molla Alemayehu, Ahmed Bedru Omer, Pedro Guillermo Suarez, Yewulsew Kassie, Anteneh Kassa, Daniel Gemechu, Degu Jerene

**Affiliations:** 1 United States Agency for International Development Eliminate Tuberculosis Project, *Koninklijke Nederlandse Centrale Vereniging* Tuberculosis Foundation, Addis Ababa, Ethiopia,; 2 United States Agency for International Development Eliminate Tuberculosis Project, Management Sciences for Health, Addis Ababa, Ethiopia,; 3 National Tuberculosis and Leprosy Program, Federal Ministry of Health, Addis Ababa, Ethiopia,; 4 Management Sciences for Health, Arlington, Virginia, United States of America,; 5 United States Agency for International Development, Addis Ababa, Ethiopia,; 6 *Koninklijke Nederlandse Centrale Vereniging* Tuberculosis Foundation, The Hague, The Netherlands.

**Keywords:** Tuberculosis, elimination, approaches

## Abstract

To end the global tuberculosis (TB) epidemic and eliminate TB, countries around the world committed to significantly expanding the scope of their efforts, including rapid uptake of new tools, interventions, and strategies, and envisioned a world free of TB. Between 2010 and 2020, Ethiopia experienced a 5% average annual decline in TB incidence. However, at that current rate, ending the TB epidemic (<10 TB cases/100,000 population) may not be possible soon. As a high TB and TB/HIV burden country, Ethiopia’s TB epidemic is characterized by a high rate of transmission in the general population and hard-to-reach areas and progression of latent TB infection (LTBI) rather than cross-border migration. Studies suggest that a combination of interventions, such as intensive household screening with TB preventive therapy, has the potential to significantly decrease the incidence of TB. The feasibility of reducing the population-level TB incidence by a combination of interventions in Ethiopia is unknown. Based on the World Health Organization’s TB elimination framework and the END TB strategic documents and previously published reviews in TB elimination we conducted a narrative review to summarize and estimated the effect of a combined intervention package (community-based TB screening for active case finding and TB and LTBI prevention and treatment among high-risk groups like household and close contacts). The projected annual decline of TB incidence was above 16%. With this level of impact and nationwide scale-up of the interventions, Ethiopia aligns well with ending the TB epidemic before 2035 and shifting toward TB elimination in the foreseeable future. In the Ethiopia setting, we recommend future studies generating evidence on the impact of the combination intervention package to reduce TB incidence in Ethiopia, which is aiming to shift from control to TB elimination.

## Introduction

Despite the curable and preventable nature of tuberculosis (TB), the disease continues to be a public health problem globally, affecting an estimated 10 million people annually and causing 1.3 million deaths among HIV-negative people and an additional 214,000 deaths among HIV-positive people [1]. The African region accounts for nearly one-third of the estimated global burden of TB [2]. The global TB incidence is declining at 2% per year [1], and without further intervention, it is estimated to continue at the same rate. With the unprecedented impact of COVID-19, the annual rate of decline in TB incidence reversed to the level seen eight years ago [1].

In the recent past, African countries experienced a progressive decline in TB incidence [1,2]. However, further advancements in ending the epidemic and ultimately achieving TB elimination have slowed [3]. To end the global TB epidemic, countries around the world committed to significantly expanding the scope of their efforts in the areas of early and universal access to diagnosis and treatment, strengthening government leadership in multisectoral actions against TB, and research and innovations [3]. The long-term vision is a world free of TB, and the strategic goal is to end the global TB epidemic by 2035, defined as a global incidence of fewer than 100 cases/million populations. This will require a 95% reduction in the number of deaths due to TB and a 90% reduction in the incidence of TB [4].

In 2014, the World Health Organization (WHO) introduced a framework to eliminate TB in low-incidence countries [3]. The WHO highlighted eight priority action areas to reach TB elimination. Addressing key affected populations, active TB screening and latent TB infection (LTBI) identification in high-risk groups, optimizing multidrug-resistant (MDR)-TB prevention and care, and investing in research and new tools were among the key intervention areas. This framework aimed to achieve pre-elimination of TB (<10 TB cases/million) in 2035 and elimination (<1 TB case/million) in 2050 in countries that are approaching the low TB incidence level [5]. In high-resourced countries such as the United States [6] and European countries [7], the comprehensive strategies designed to eliminate TB have achieved significant results, although they have been challenged by TB outbreaks among cross-border migrants, emerging immune-compromising conditions, and inadequate TB infection control [8].

Although TB elimination strategic frameworks had been prepared by WHO for low TB incidence countries, they should also be considered by countries with an intermediate and steadily decreasing TB incidence (i.e. <50 cases/100,000 population) [9]. The 2020 WHO *Consolidated Guidelines on TB: TB Preventive Therapy* recommend implementing all interventions at maximum potential and are now applicable to any country, including high TB incidence countries [10]. For example, in high TB burden countries such as India, Pakistan, Vietnam, and Bangladesh, a ZERO TB Initiative was launched to support cities, districts, and islands that are committed to achieving a rapid reduction in the number of people suffering from TB. This initiative calls for coalitions among local governments, businesses, and civil society; uses the comprehensive Search-Treat-Prevent approach; and focuses TB prevention and care on households (HHs), the places where people seek care, and where people work [11].

Tuberculosis elimination introduced in an integrated manner, including in rural and urban settings in resource-constrained and high TB burden countries, may accelerate the decline in TB incidence. However, an intensified and accelerated TB case reduction strategic framework has never been used or studied in a low-income and high TB burden country like Ethiopia. There is a need to develop a context-based intervention package that can lead to a significant decline in TB incidence [12-14], which will later help achieve the TB elimination goal while passing through the pre-elimination phase, where TB elimination is conceivable and could be reached [15]. National TB programs (NTPs) also need to start making changes to their thinking, organization, and design of interventions, from control to elimination [16,17].

This review aims to present evidence to support the combination intervention package to reduce TB incidence in Ethiopia-country aiming to shift from control to TB elimination in the near future. The article will address critical gaps in much-needed country-specific plans and strategies for TB elimination programs, which other countries with similar settings may learn from.

## Methods

The basis for this narrative review is WHO´s TB elimination framework [1]; the END TB strategic documents beyond 2015 [18]; previously published reviews assessing progress of TB elimination at the regional or national levels [7,14-16,18-22]; and the NTP and national strategic plan in Ethiopia [23,24]. A writing group comprising the Ethiopian Federal Ministry of Health (FMoH); NTP; Regional Health Bureaus; and several US Agency for International Development (USAID)-funded projects´ lead organizations, such as Management Sciences for Health (MSH) and *Koninklijke Nederlandse Centrale Vereniging* (KNCV) TB Foundation, drafted the review.

Based on progress made in the last 10 years and literature estimates of the effect of combined intervention (community-based TB screening for active case finding (ACF), TB and latent TB infection (LTBI) prevention and treatment among high-risk groups), the annual decline of TB incidence was projected through 2035 and beyond. The main factors included in the projection and estimates were: 1) the routine program performance as a baseline, e.g. annual TB incidence taken from the annual WHO report; 2) the number of contacts per index TB case; 3) risk of TB among contacts; 4) proportion of potentially preventable TB among household contacts; 5) tuberculosis preventive therapy (TPT) coverage among household contacts and PLHIV; and 6) estimated TB prevention with efficacy of TPT.

The draft review was also presented as a panel discussion at the 15^th^TB Research Annual Conference in Addis Ababa March 22-23, 2021, and feedback was solicited on the prospects for TB elimination and potential combination of approaches that we present in this review.

## Results

**Tuberculosis and TB/HIV trend in Ethiopia:** despite significant strides made in controlling TB, Ethiopia remains among the top 30 high TB burden countries. According to the WHO Global TB Report 2021, the rates of national TB incidence, TB in HIV, TB/HIV co-infection, and MDR-TB were 132/100,000; 8.6/100,000; 6.5%; and 1.4/100,000, respectively [1]. Approximately 29% of the estimated TB cases were reported to be missing (Table 1). Of those notified to the NTP [1], cases predominated in the younger population, and 70% of notified cases were in the age group of 15-54 years [25]. TB prevalence at the sub-national level was reported to vary from 90-256/100,000 population [25]. Nevertheless, Ethiopia is among a few high TB burden countries that demonstrated a consistent decline in the TB incidence rate, from 369/100,000 population in 1990 to 132/100,000 population in 2020. Similarly, the TB-related mortality rate declined from 89/100,000 in 1990 to 17/100,000 in 2020 [1].

**Table 1:** the current gaps between global priority indicators and targets for monitoring the implementation of the end TB strategy by 2025

Priority indicators	Status in 2020	Targets by 2025	Gap to target
Treatment coverage	71.0%	≥90%	19%
TB treatment success rate	90.0%	≥90%	0%
Preventive treatment coverage			
Children under 5 years of age	31%	≥90%	59%
People living with HIV	42%	≥90%	48%
Tuberculosis affected households facing catastrophic costs	Not available	0%	Not available
Uptake of new diagnostics	36%	≥90%	54%
Uptake of new drugs	Not available	≥90%	Not available

**Figure 1:**
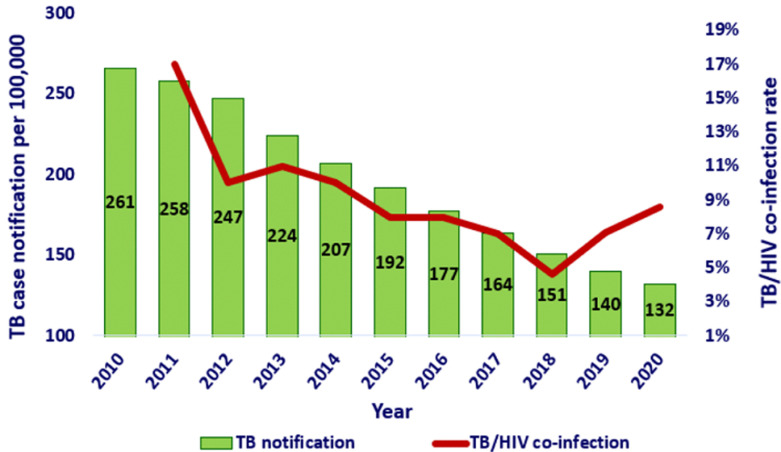
tuberculosis and TB HIV trends in Ethiopia (2010-2020)

In the last six years, through concerted efforts of key stakeholders, the TB incidence rate declined from 192/100,000 population to 132/100,000 population, which is a 5% decline on average annually (Figure 1) [1,26]. Although Ethiopia met one of the end TB milestones (at least 20% TB incidence reduction compared to 2015) [18], with this current rate of decline, ending the TB epidemic (<10 TB cases/100,000 population) and reaching the targets for ending TB (to reduce TB deaths by 95% and to cut new cases by 90% between 2015 and 2035) may not be possible in the near future, as there are considerable gaps between the expected target and the current status (Table 1). Therefore, scale-up of working interventions such as HH contact tracing, ACF, and TB preventive strategies on a larger and more sustainable scale is essential.

**Eliminate tuberculosis components in Ethiopia:** the cornerstones of the WHO-recommended end TB strategies are universal health coverage with innovative approaches that combine diagnostic, treatment, and preventive interventions [4]. Recent surveys conducted in Europe demonstrated that the majority of countries evaluated do not have all of the interventions in place to reach elimination [7]. Furthermore, the studies in Europe emphasized the importance of studying countries that are able to demonstrate that TB elimination can be reached [7,19,20]. This, however, remains to be seen as there are limited data demonstrating the impact of combination TB elimination interventions.

**Figure 2:**
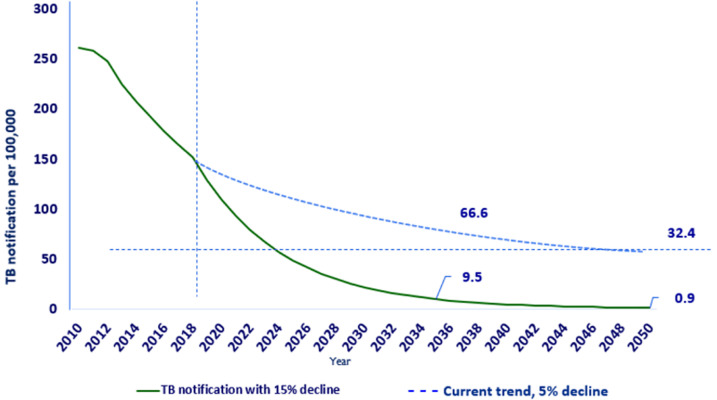
tuberculosis notification trend in Ethiopia, current 5% versus estimated 15% annual decline

To reduce the population-level incidence of TB in Ethiopia, it is essential to have context-based large-scale interventions aimed at interrupting further transmission and averting future cases. Households contact screening, intensive (mass) community-based TB screening for ACF in geographic areas with poor access to diagnostics in high TB burden settings, and identifying individuals with LTBI on a large scale are key to eliminating TB in Ethiopia. According to our estimates and projections, ending the TB epidemic before or by 2035 Ethiopia is possible if TB incidence declines by an annual factor of 15% or more from the current incidence of 132/100,000 population (Figure 2). This requires highly effective strategies to reduce TB transmission among populations at risk of developing TB, such as HH contacts and people living with HIV (PLHIV). Moreover, to halt the TB epidemic, ACF and prompt initiation of the correct treatment are essential components of TB elimination strategies [27]. Details on the potential effects of intensive community-based TB screening, HH contact investigation, and tuberculosis preventive therapy (TPT) among HH contacts and PLHIV within the Ethiopian context are described below.

**Community-based screening and active case finding:** intensive (mass) community-based screening is only recommended among subpopulations with poor access to health care; those living in poor areas and remote areas; and those associated with other risk factors (e.g. prisoner, migrant, refugee, homeless) [28] (Table 2). Otherwise, indiscriminate mass screening is not the WHO-recommended strategy for case finding due to its low benefit (the yield ranges from 0.1% to 0.7% [28]). Data from 18 prevalence surveys demonstrated that in many settings, more than half of the prevalent TB cases in a community were undiagnosed [28]. For similar reasons, the TB prevalence survey in Ethiopia suggested the need to strengthen community-based screening for early detection and treatment of cases to reduce TB transmission in the community [25]. Therefore, targeting entire communities (i.e. subpopulations with poor access to health care) through mass screening is critical in a high TB burden country such as Ethiopia. The feasibility and impact of community-based screening have been studied in the recent past in Southern Ethiopia, where Yassin *et al*. demonstrated a close to doubling of the TB incidence rate, from 102 cases/100,000 population (95% confidence interval, CI: 99.1-105.8) before implementation to 177 TB cases/100,000 population (95% CI: 172.6-181.0) after community intervention, and improved treatment outcomes for bacteriologically confirmed TB (77% [95% CI: 75.0-78.8] to 93% [95% CI: 91.8-94.2]) before and after community intervention (the package included advocacy, training, engaging stakeholder and community member, and ACF using house-to-house visits and TB screening by female extension health workers) [29]. A randomized controlled trial by Datiko *et al*. found higher mean TB case-detection rates in the intervention communities (122.2% versus 69.4% control, p=0.001), which included increasing awareness of TB and TB symptoms, facilitating sputum collection, and supporting treatment in the community [30]. In Ethiopia, because close to one-third of TB cases are estimated to be missed [1], a focused community-based screening in geographic areas with a high TB burden may help find missed TB cases early, thereby reducing TB transmission, and early treatment may improve treatment outcomes. To guide the targeted intervention, Ethiopia may prioritize regions, zones, and districts with TB incidence above the current WHO estimates, which is 132,000/100,000 [1], those with poor access to health facilities. In addition, symptom screening with chest X-ray regardless of symptom should be extended to high-risk groups such as PLHIV, health care workers, prisoners, migrants, patients with Diabetes Mellitus, children < 5 years of age and HH, and close contacts [10].

**Table 2:** key intervention areas for accelerated TB incidence reduction in Ethiopia

Type of intervention	Standards of care	Key action area	Effect on TB incidence
1.0 Community- based TB screening and active case finding	- Health extension workers ask for TB symptoms during routine home visits.	- Annual mass screening among subpopulations with poor access to health care, such as those living in poor and remote areas.	- Minimize the missed TB cases (29%).
	- Symptom-guided TB screening, Xpert MTB/RIF for all presumptive TB patients.	- Symptom-guided TB screening, chest X-ray, and Xpert MTB/RIF for all presumptive TB patients.	
		- Simultaneous symptom screening and digital X-ray with artificial intelligence (X-ray for all high-risk groups, such as PLHIV, HCWs, prisoners, DM, and household and close contacts) regardless of TB symptoms.	- Maximize TB case finding and narrow the gap between the reported and estimated incident TB cases.
			- Maximize opportunities for TB preventive therapy among those who screened negative for TB.
2.0 Tuberculosis preventive therapy	- Eligible PLHIV offered TPT in a routine setting.	- Eligible PLHIV offered TPT in a routine setting; mobilize indigenous community structures (e.g., Iddirs) to increase TPT-seeking behavior.	- Maximize coverage to 90% from the current 42%.
	- Under 5 years of age HH contacts followed passively.	- Under 5 years of age HH contacts actively followed up by mobilizing indigenous community structures (e.g., Iddirs) to increase TPT-seeking behavior.	- Maximize coverage to 90% from the current 31%.
		- Active follow-up of HH and close contacts, health care workers, prisoners, and DM by mobilizing indigenous community structures (e.g., Iddirs) to increase TPT-seeking behavior.	- Maximize coverage to 90%; the current status not known.
3.0 Contact investigation	- Routine, passive, and prospective contact investigation.	- Active, prospective, reverse, and retrospective or follow-up contact investigations.	- Minimize the missed TB cases or narrow the gap between the reported and estimated incident TB cases.

**Tuberculosis preventive therapy in Ethiopia:** in Ethiopia, TPT has been recommended for high-risk groups, particularly PLHIV and children under the age of 15 who are HH contacts of infectious TB cases, to reduce the risk of progression to active TB disease. Although the recommendations have been in national TB and HIV guidelines for more than a decade, the implementation of TPT for either of the eligible priority risk groups has been low (42-49%) [1,2]. Several challenges and barriers related to the capacity of health care providers, consistency in quality of TB screening, passive contact tracing, issues of adherence with increased pill burden, and concerns about potential drug resistance with isoniazid monotherapy have been attributed to the protracted progress of TPT implementation in the country. In 2020, 15,635 PLHIV newly enrolled in HIV care and 42% were on TPT, and among children under 5 years of age who were contacts of bacteriologically confirmed pulmonary TB cases only 31% were initiated on TPT (Table 1). This is far from what the country intends to achieve [1].

### Tuberculosis preventive therapy among high-risk groups

**Tuberculosis preventive therapy among households contacts with index tuberculosis cases:** on average, among the estimated 10 contacts identified for each person with infectious TB, 30% to 51.4% are found to have LTBI, and 4% to 5% of contacts develop active TB [31,32]. Of the contacts who will ultimately have TB disease, approximately 75%, 81%, and 92% develop TB disease in the first three months, six months, and one year after exposure, respectively [31,32]. From the Sidama region in Southern Ethiopia, Yassin *et al*. in 2020 reported that from 1,517 HH contacts of 344 index cases who were visited and screened for TB and followed up for a median of 37 months, 5% (77/1,517) developed TB during 4,713 person-years of follow-up with an estimated TB incidence of 1,634 (95% CI: 1,370-2,043) per 100,000 person-years of follow-up, which is much higher than the estimated TB incidence for the general population in Ethiopia of 210/100,000 [33].

**Figure 3:**
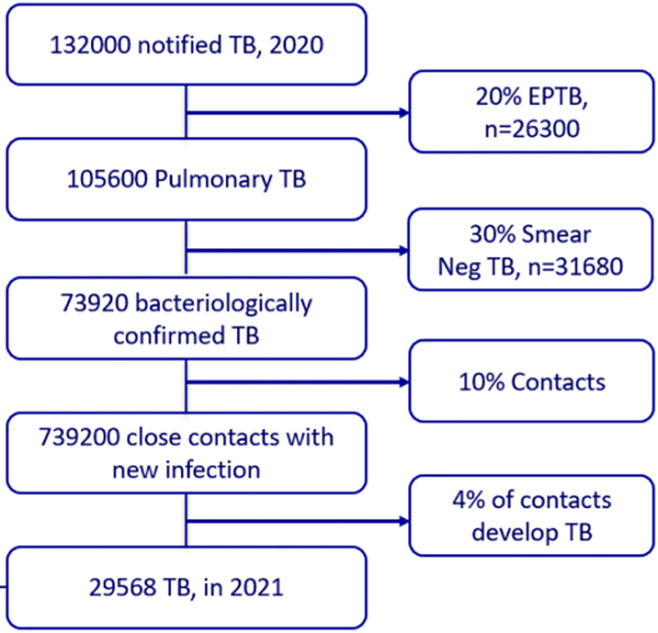
estimated TB among contacts of potential bacteriologically confirmed index TB cases in 2021

In the context of Ethiopia, among the 132,000 notified TB cases in 2020, 29,568 new TB cases were estimated in 2021 to come from the pool of close contacts, representing 22.4% of the notified TB cases. In this estimate, the assumptions included 20% extrapulmonary TB, 30% bacteriologically unconfirmed TB (smear negative), the remaining bacteriologically confirmed TB infecting 10 other people, and 4% of contacts developing active TB (Figure 3) [32]. To avert the active TB cases coming from households and close contacts, if the resource allows the programmatic approach for Ethiopia might be bi-annual or annual screening among households and other high-risk groups. Asymptomatic individuals after screening might be offered TPT.

In earlier trials conducted among HH contacts, a 74% to 77% reduction in TB cases was achieved after one year of treatment with isoniazid preventive therapy [34,35]. Tuberculosis preventive therapy, especially with higher coverage when combined with other prevention and treatment strategies, will contribute to TB elimination [36-39]. With the implementation of TPT among HH contacts, there is great potential to avert a substantial number of incident TB cases in Ethiopia. With TPT implementation among HH contacts and an assumption of 50% efficacy of TPT (i.e. prevent the development of TB by 50%), more than 18,000 TB cases are estimated to be averted in one year, which translates to a reduction of 16 TB incidence/100,000 population, which is twice the current annual average decline of eight TB incidence/100,000 population.

**Tuberculosis preventive therapy among people living with HIV:** in a recent systematic review, the effectiveness of TPT in risk reduction for TB among PLHIV was well demonstrated, with 33% overall and 64% among those who were tuberculin skin test positive [10]. In 2019 and 2020, there was suboptimal uptake of TPT (42 and 49%) among HIV-positive newly enrolled cases, and 10,000 HIV-positive cases were reported as incident TB [1,2,40] in Ethiopia. To achieve an optimal reduction of incident TB among HIV-positive people, TPT coverage needs to be scaled up. With the scale-up of TPT to more than 90% and 50% efficacy of TPT, close to 5,000 HIV-positive TB cases are estimated to be averted, which translates to 4.5 TB incidence per 100,000 populations in one year.

**Tuberculosis preventive therapy among other high-risk groups:** because of an increased risk for progression to active TB, healthcare workers, prisoners, homeless people, and immigrants are considered to be high-risk groups and are prioritized for systematic TB screening, testing, and treatment of LTBI [10]. Because evidence of the benefit in people with diabetes is limited, systematic testing and treatment is not recommended by WHO. However, depending on the settings (i.e. with an increased risk for progression to active TB), countries may consider offering TPT among people with diabetes [10]. In Ethiopia, the prevalence of diabetes among people 18 years of age and above is increasing [2].

**Multidrug-resistant tuberculosis (MDR-TB) prevention and care:** for many years, Ethiopia was one of the 30 high MDR/RR-TB burden countries, but in 2021 it was removed from the list [41]. The country cites political commitments; expansion of laboratory, clinical, and community-level services; and significant in-country and global collaborative efforts for this achievement. During the implementation period of the just-ending tuberculosis and leprosy national strategic plan (TBL-NSP), services have expanded, effectively allowing decentralized access to diagnostics, including for TB culture and drug susceptibility and testing, and treatment of drug-resistant tuberculosis (DR TB) in peripheral settings [24].

Nonetheless, MDR-TB in Ethiopia remains a significant challenge and needs to be a focus to work toward ending the TB epidemic and elimination. A significant proportion of MDR-TB cases who continuously transmit the disease are not caught by routine care. In 2019, only 47% of the estimated MDR/Rifampicin-Resistant TB cases were enrolled in treatment. The same report stated a 75% treatment success rate [2]. Highly sensitive case-finding strategies and high-quality patient management is one priority area to improve DR-TB care. The long treatment duration with highly toxic drugs imposes significant challenges on adherence to treatment. To improve our case finding, we must conduct contact screening for all bacteriologically confirmed TB/DR-TB cases and enroll all patients in treatment. Optimal DR-TB case management and improvement of treatment outcomes need universal implementation of active drug safety monitoring and patient-centered support. Both first- and second-line drug susceptibility testing coverage should be scaled up for appropriate regimen design and prompt assignment of patients to effective treatment to halt continuous transmission.

## Discussion

From our assessment and projection reaching TB elimination by 2050 in Ethiopia is too ambitious, given the efficacy of the current tools and health service delivery [38]. Globally, notwithstanding the progress made toward meeting Millennium Development Goals (MDGs) and the post-MDG WHO TB elimination framework, only four countries, Antigua and Barbuda, Barbados, Montserrat, and Niue and San Marino, has ever reached TB elimination. In 2019, 54 countries had achieved ending the TB epidemic (<10 cases/100 000 population/year). Morocco is the only country in Africa reaching such a low incidence of TB; the rest are mostly in the Americas and European region [2].

Unlike most countries in the WHO African region, Ethiopia has achieved the 2020 milestone toward ending the TB epidemic targets, which is at least 20% reduction in the absolute number of TB cases between 2015 and 2020 [1]. However, with the current rate, ending the TB epidemic in Ethiopia may not be possible soon. Thus, the rate needs to decline rapidly, with a 15% average annual decline to end the epidemic and a 20% average annual decline to eliminate TB before or in 2050, which requires a considerable commitment.

Intensified research and innovation in line with TB elimination is one of WHO´s eight priority action areas [4]. The global strategy emphasizes that countries need to study their TB epidemic and design customized combination interventions if TB control is to shift to elimination [12-14,42]. To that effect, many countries, particularly in Europe, are working on a context-based TB elimination framework [7,42,43]. In this review, end TB strategies tailored for Ethiopia for a potential accelerated TB incidence decline using a high-impact combination intervention are presented.

The experience from low TB incidence countries [15] shows that the combined TB interventions recommended by WHO in 2014 have been implemented. These countries, however, were challenged by TB transmission in high-risk groups, such as migrants from high TB burden countries [15]. For example, 41% of Oman´s population were migrants from high-incidence countries and accounted for 60% of the annual TB cases [13]. The ZERO TB initiative in the cities and islands of Asia-Pacific countries applied a Search-Test-Treat approach, putting in place a set of TB prevention and control interventions [11]. Data from previous experience suggest that a remarkable reduction in TB incidence may not be possible with a single intervention unless a combination of preventive, diagnostics, treatment, and follow-up interventions are in place. Although Suárez *et al*. reported that the widespread application of directly observed treatment, short course (DOTS) in Peru in the 1990s was associated with a marked and sustained decline in TB incidence [44], subsequent evidence from Brazil and New York City, however, suggests that multiple interventions, rather than DOTS alone, are likely to be associated with an accelerated decline in TB incidence [45,46]. Hence, a combined approach of case finding, active contact racing, identifying high-risk and treatment of LTBI, and improved surveillance are essential.

For Ethiopia, there are a number of opportunities to assist in accelerating the decline in TB incidence: (1) using local evidence, we know the working interventions to end the TB epidemics; (2) together with the stakeholders and partners the NTP is launching a quasi-experimental study aiming to demonstrate accelerated TB decline with a combination intervention, the result of which may help define a pathway toward TB elimination that may be scaled up nationally; (3) Ethiopia has a solid TB control program, although an elimination plan has not been formalized, the NTP is committed to TB elimination and is actively participating in the experimental study; (4) to increase access to diagnosis, Xpert MTB/RIF systems are decentralized at the woreda (district) level, and line probe assays are available at central and regional laboratories; (5) TPT, including the shorter three-month isoniazid and rifapentine (3HP) regimen, is included in the national guidelines; and (6) the shorter oral regimes for DR-TB are also adopted in the country and are being implemented.

A lot of uncertainty remains in the following areas, which are considered major challenges in the context of Ethiopia: (1) High-level commitment. There is a need for a national TB elimination plan, supported by high-level ministerial commitment. India is a good example of this, whereby TB elimination is given ministerial attention with regular follow-up [2]. (2) Funding and sustainability. The government expenditure on health is not optimal. The 2021 evaluation report indicated that about half of the resources available to implement the national TB strategic plan were from donor contributions, and the rest were from the government budget [47]. Per the WHO 2020 global report, the TB-specific funding gap remains at more than 50% (USD 47 million) [2]. To achieve end TB strategies, a TB-specific budget needs to be allocated, particularly for maintaining quality health service that should be free for TB patients. (3) Sub-optimally operating infrastructure and health systems. Ethiopia has major human resource challenges, including a shortage of health care workers, urban/rural and regional disparities, poor motivation and retention, and suboptimal performance [48]. The shortage of laboratory professionals, particularly in rural health facilities, is a challenge. Further, the number of radiologists is small [24]. Staff improvement both in quality with adequate training and rational coverage of health facilities with enough staff to deliver TB services is of paramount importance, and this is also dependent on a well-funded environment. (4) Intersectoral collaboration for poverty alleviation and improvement of undernutrition and poor housing condition; and (5) impact of COVID-19. Ethiopia is not exempt from the effect of the pandemic, which is even more challenging with ongoing instability in some part of the country. Globally, the success of TB incidence reduction has been reversed by eight years (to 2012). Tuberculosis screening strategies need to be coordinated with COVID-19 activities.

Our review has some limitations. First, as a narrative review there was not a strict protocol or inclusion and exclusion criteria followed and the methods used for the literature review was dependent of the objective of the review focus area set by the authors. Second, the design of the studies included varies from clinical trial to programmatic and operational studies that brings a difference in designs in study settings, selection of population studies, data quality. Notwithstanding that there were limited body of knowledge around TB elimination, particularly in African settings, the authors attempted to include all available relevant published data focused on TB elimination globally, and those conducted in African region, including Ethiopia. We presented evidence to support the combination intervention package to reduce TB incidence in Ethiopia, which is aiming to shift from control to TB elimination in the near future. This article attempted to address critical gaps in much-needed country-specific plans and strategies for TB elimination programs, which other countries with similar settings may learn from.

## Conclusion

Although there is a lot of global experience with TB elimination trials, most evidence is concentrated in developed countries with low TB incidence. However, evidence from low-income countries like Ethiopia is also worth generating. Therefore, enhancing research in countries with a substantial TB burden is important for TB epidemic reduction or elimination. In Ethiopia, the planned study may assist in developing and implementing a novel TB elimination framework for the TB program, building on the current success of the NTP. With the current rate of TB incidence reduction in Ethiopia, the milestones of end TB - pre-elimination and elimination - would not be achieved. However, if the defined TB elimination packages are introduced and enhanced, it is possible to achieve the elimination rate with the annual TB reduction rate of 16%. Hence, it is recommended to introduce the combination intervention packages in Ethiopia to develop the national TB elimination framework so that the country might shift from a control to an elimination TB program.

**Funding:** this research has been supported by the US Agency for International Development (USAID) through Management Sciences for Health (MSH) under Cooperative Agreement No. 72066320CA00009 and the KNCV Tuberculosis Foundation.

**Disclaimer:** the findings and conclusions in this report are those of the authors and do not necessarily represent the official position of the funding agencies. References in this manuscript to any specific commercial products, process, service, manufacturer, or company does not constitute its endorsement or recommendation by the U.S. government, MSH or KNCV Tuberculosis Foundation.

### What is known about this topic

- The 2020 WHO Consolidated Guidelines on TB: TB Preventive Therapy recommend implementing all interventions at maximum potential and are now applicable to any country, including high TB incidence countries;
- Multiple interventions, rather than DOTS alone, are likely to be associated with an accelerated decline in TB incidence;
- Between 2010 and 2020, Ethiopia experienced a 5% average annual decline in TB incidence; however, at that current rate, ending the TB epidemic (<10 TB cases/100,000 population) may not be possible soon.

### What this study adds

- This review showed the evidence for effect of a combined intervention package of community-based TB screening for active case finding and TB and LTBI prevention and treatment among high-risk groups (e.g. household [HH] and close contacts, health care workers);
- With the combination intervention the projected annual decline of TB incidence was above 16%, and with this level of impact and nationwide scale-up of the interventions, Ethiopia aligns well with ending the TB epidemic before 2035 and shifting toward TB elimination in the foreseeable future;
- This review sets a stage for other low- and middle-income countries to consider combination intervention if they are to achieve TB elimination.
